# The Effect of *UGT1A1* Promoter Polymorphism in the Development of Hyperbilirubinemia and Cholelithiasis in Hemoglobinopathy Patients

**DOI:** 10.1371/journal.pone.0077681

**Published:** 2013-10-30

**Authors:** Suad AlFadhli, Hassan Al-Jafer, Mays Hadi, Mashael Al-Mutairi, Rasheeba Nizam

**Affiliations:** 1 Department of Medical Laboratory Sciences, Kuwait University, Jabriya, Kuwait; 2 Al-Amiri Hospital Medical Laboratory, Ministry of Health, Kuwait City, Kuwait; University Medical Center Hamburg-Eppendorf, Germany

## Abstract

Present study was aimed to explore the effect of (TA)_n_ *UGT1A1* gene promoter polymorphism on bilirubin metabolism, bilirubinaemia, predisposition to cholelithiasis and subsequent cholecystectomy, in Sickle-Cell Anemia (SCA) and beta-Thalasemia major (bTH) in Kuwaiti subjects compared to other population. This polymorphism was analyzed and correlated to total bilirubin and cholelithiasis in 270 age, gender, ethnically matched subjects (92 bTH, 116 SCA and 62 Controls) using PCR, dHPLC, fragment analysis and direct sequencing. Four genotypes of *UGT1A1* were detected in this study (TA6/6, TA6/7, TA6/8 and TA7/7). (TA)6/8 was found only in four individuals; hence it was not included in the analysis. There was a statistically significant association of genotypes with serum total bilirubin levels in both bTH and SCA groups *(p<0*.*001)*. Subjects with (TA)7/7 had the highest total serum bilirubin level (178.7±3.5 µmole/l). A significant association was observed between allele (TA)7 and cholelithiasis development *(p = 0*.*0001)*. The 40%, 67.5% and 100% of SCA with (TA)6/6, (TA)6/7 and (TA)7/7 respectively developed cholelithiasis and were subsequently cholecystectomized. Our results confirm *UGT1A1* (TA)7 allele as one of the factors accounting for the hyperbilirubinemia and cholelithiasis observed in SCA and bTH.

## Introduction

Bilirubin is a toxic metabolite, predominantly resulting from the turnover of hemoglobin. Uridine diphosphate glucuronosyltransferase 1A1 (*UGT1A1*), the A1 isoform encoded by the bilirubin UDP glucuronosyltransferase 1 family (*UGT1*), catalyzes glucuronidation of bilirubin in humans through the formation of intermediate derivatives mono- and diglucoronides [1]. The Elevation of unconjugated bilirubin due to inadequate bilirubin glucoronidation is associated with the accumulation of serum bilirubin, [2] this unconjugated hyperbilirubinemia results from decreased activity of *UGT1A1* enzyme to approximately 30% of normal levels [3].

*UGT1A1* is encoded by *UGT1A1* gene, which consists of five exons and is a part of the *UGT1A* locus on chromosome 2q37 [4], [5]. Polymorphisms in *UGT1A1* gene promoter have been shown to affect transcriptional efficiency, strongly influencing bilirubin metabolism and clearance [6]. The wild type promoter contains an A (TA) nTAA sequence with (TA)6 repeats, while a less frequent allele contains extended repeat sequence (TA)7. The homozygous genotype of the latter allele was associated with unusually high levels of bilirubin and a significantly increased frequency of gallstones and gall bladder disease [6]–[8]. The (TA)7 allele has also been associated with increased bilirubin levels in apparently healthy individuals. Two other alleles, (TA)5 and (TA)8, also been identified, primarily in individuals of African descent [9].

Beta Thalassemia major is a quantitative problem with a genetic defect that results in reduced rate of synthesis of the two beta globin chains causing severe anemia, often through mutations in regulatory genes. On the other hand, sickle-cell anemia (a hemoglobinopathy) is a qualitative problem of synthesis of an incorrectly functioning globin with mutation of the sixth amino acid valine to glutamine. The High levels of erythrocyte destruction in patients with SCA and to a lesser extend in bTH result in chronic hyperbilirubinemia. A significant proportion of patients are prone to cholelithiasis due to high biliary concentration of unconjugated bilirubin, which tends to coprecipitate with calcium in the gall bladder lumen. Cholelithiasis, by promoting cholecystitis and choledocholithiasis, is responsible for high levels of morbidity in hemoglobinopathy patients [2] and elective cholecystectomy is therefore recommended for patients developing this complication [10]. The coinheritance of aforementioned hematological diseases and *UGT1A1* gene promoter A (TA)_ 7TAA_ polymorphism will probably increase the risk of developing cholelithiasis in such patients [2], [6]–[8].

No data have so far been reported about the prevalence of *UGT1A1* (TA)*_n_* polymorphism in the Kuwaiti population except for one article screened the Kuwaiti G6PD patients for *UGT1A1* (TA)*_n_* polymorphism without including healthy control [11]. Herein we carried out a population study to screen the frequency of different (TA) repeats in Kuwaiti population compared to other ethnicities. We have further analyzed the correlation between this polymorphism and hyperbilirubinemia and the prevalence of cholelitheasis in the tested SCA and bTH patients.

## Results

Screening region of interest revealed the existence of four genotypes of *UGT1A1* (TA)*_n_* polymorphism in Kuwaiti population (6/6, 6/7, 6/8 and 7/7). Genotype (TA) 6/8 was detected only in 4 out of 236 tested cases; hence it was excluded from the analysis. Genotype (TA) 6/7 was predominant in both tested cases (≥63%) and controls (55%) while (TA) 7/7 was found to be rare (<6%) in both cohorts (table 1). Observed allele and genotype frequencies of *UGT1A1* (TA)*_n_* polymorphism failed to show any significant difference between the tested subjects *(p>0*.*05)* with respect to age or gender.

**Table 1:** Allele and genotype frequencies of *UGT1A1* promoter *polymorphism* in β-Thalassemia, Sickle Cell Anemia and Healthy Controls (# total Bilirubin measured in µmol/L).

	bTH (n = 70)		SCA (n = 104)		HC (n = 62)	
Genotype	*Freq n (%)*	*Total Bilirubin*	*Freq n (%)*	*Total Bilirubin*	*Freq n (%)*	*Total Bilirubin*
6/6	18 (27)	20±9	30 (29)	30.1±13.1	26 (42)	13.9±1.38
6/7	44 (63)	28.5±17.7	68 (65)	58±26.4	34 (55)	15.2±2
6/8	4 (5)	28.5±15	0	–	0	–
7/7	4 (5)	71±43	6 (6)	178.7±3.5	2 (3)	15
**Allelotype**	***Freq n (%)***		***Freq n (%)***		***Freq n (%)***	
6	84 (59)		124 (60)		86 (67)	
7	52 (38)		84 (40)		38 (33)	
8	4 (3)					

n = Number of samples of a particular genotype/allelotype.

Freq = Frequency.

**Figure 1:**
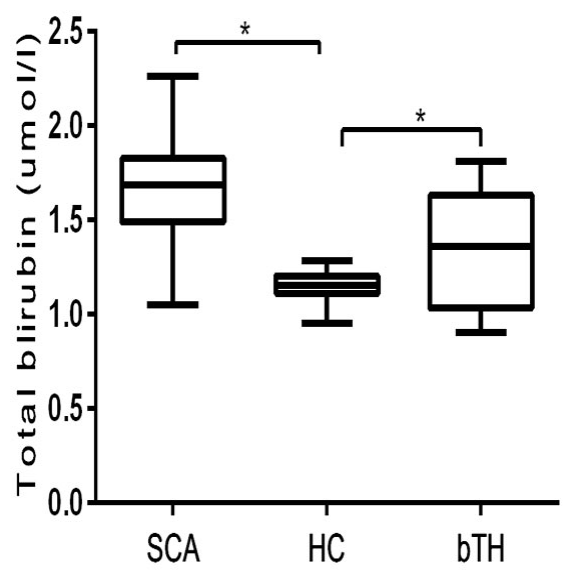
Represents serum total bilirubin in SCA, HC and bTH. A significant association of serum total bilirubin was observed with bTH *(p = 0.001)* and SCA subjects *(p<0.0001)* when compared individually to healthy control by ANOVA test. *Indicates Significance p<0.05.

To evaluate the association of *UGT1A1* (TA)*_n_* polymorphism with hyperbilirubinemia, serum total bilirubin of each tested subjects were detected. A significant association of average serum total bilirubin was observed with bTH and SCA subjects (p<0.05). Average serum total bilirubin was higher in SCA (57.12±39 µmol/L, *p<0*.*0001*) than in bTH (28.87±20.4 µmol/L, *p = 0*.*001*), when compared individually to healthy control (14.34±2.20 µmol/L) (figure 1). As shown in table 1 subjects with genotype (TA)7/7 had the highest levels, while (TA)6/7 had intermediate levels and the lowest level was found in genotype (TA)6/6. The average serum total bilirubin was significantly higher in bTH with 6/6 *(p = 0.0014)* and 6/7 genotype *(p = 0.0001)* and SCA with 6/6, 6/7 and 7/7 genotype *(p = 0.0001)* when compared to individually to the healthy control with respective genotype. There was a significant association between the various genotypes of *UGT1A1* (TA)*_n_* polymorphism and the serum total bilirubin levels in both bTH and SCA subjects (*p*<0.001), however no significant difference was observed based on gender.

All recruited SCA cases (104) underwent liver/biliary ultrasound scans and their data was available. A 67.3% of 104 of the tested SCA patients had gallstone disease. SCA patient having cholelithiasis had significantly higher serum total bilirubin (66.5±44.7 µmol/L), conjugated bilirubin (16.3±17.6 µmol/L) and unconjugated bilirubin concentrations (50.7±29.6 µmol/L) than the patients without cholelithiasis (Table 2). No significant difference in hemoglobin concentration, RBC or WBC count was observed between the tested subjects. When student’s t-test was computed, the level of total serum bilirubin and its two forms were significantly higher in patient with cholelithiasis with *p<0.05* (Table 2). The frequency of cholelithiasis in the study groups revealed; 40% SCA patients with *UGT1A1* (TA)6\6 genotype developed gallstones, 76.5% heterozygotes (TA)6\7 and 100% homozygous SCA patients with UGT1A1 (TA)7\7 had cholelithiasis (Table 3).

**Table 2:** Bilirubin levels ( µmol/L) in SCA patients with and without gallstone disease.

Serum Bilirubin	Gall Stone disease		*p-value* *
	Present (n = 70)	Absent (n = 34)	
Total Bilirubin	66.5±44.7	37.1±13.7	0.011
conjugated Bilirubin	16.3±17.6	5.4±.94	0.014
unconjugated Bilirubin	50.7±29.6	31.7±13.3	0.015

* Students t-test.

**Table 3:** Allele and genotype frequencies of *UGT1A1* promoter polymorphism in SCA diagnosed with or without gall stone.

Genotype	Cholelithiasis	Cholelithiasis	*p-value*	OR	95% CI
	Positive(n = 70)	Negative(n = 34)			
6/6	12 (40%)	18	0.0001	0.184	0.07–0.46
6/7	52 (76.5%)	16	0.006	3.25	1.37–7.69
7/7	6 (100%)	0	–		
**Allele**					
6	76	52	0.002	0.36	0.19–0.70
7	64	16		2.74	1.42–5.25

Dominant model 6/6 versus (6/7+7/7) showed increased susceptibility to cholelithiasis (p = 0.0001, OR = 5.44, 95% CI 2.17–13.59).

A significant association was observed between having allele (TA) 7 and the development of gallstone in SCA patients *(p = 0.002, OR = 2.74, 95% CI 1.42–5.25)* table 3. The development of symptomatic bilirubin gallstones requiring cholecystectomy was significantly higher for patient with the (TA) 7/7 genotype than those with the (TA) 6/6 or (TA) 6/7 genotype (*p*<0.05). An increased risk for cholelithiasis was observed among SCA when dominant genetic model was employed *(6/6 versus (6/7+7/7) p = 0.0001, OR = 5.44, 95% CI 2.17–13.59),* indicating the risk role of allele 7 in the development of gall stone.

Association of *UGT1A1* promoter polymorphism and serum total bilirubin with tested subjects was further assessed using logistics regression by adjusting for factors such as age and gender (Table 4). Analysis of *UGT1A1* polymorphism revealed no significant association with the studied diseases. However, average total serum bilirubin showed significant association with both bTH *(p = 0.016, OR = 1.036)* and SCA *(p<0.0001, OR = 1.80).*

**Table 4:** Analysis of *UGT1A1* promoter polymorphism in SCA and bTH subjects using logistic regression.

SCA	*p-value*	AOR*	95% CI
**Age**	0.98	0.998	0.87–1.14
**Gender**			
* Male*	Reference		
* Female*	0.331	2.069	0.48–8.96
**Total bilirubin**	<0.0001	1.800	1.39–2.34
**Genotype**			
* 6/6*	Reference		
* 6/7*	0.106	3.481	0.77–15.82
* 7/7*	0.965	1.146	0.003–510.6
**bTH**	***p-value***	**AOR** *	**95% CI**
**Age**	0.343	0.958	0.88–1.05
**Gender**			
* Male*	Reference		
* Female*	0.953	1.024	0.47–2.24
**Total bilirubin**	0.016	1.036	1.01–1.07
**Genotype**			
* 6/6*	Reference		
* 6/7*	0.199	1.676	0.76–3.69
* 7/7*	0.363	2.362	0.37–15.08

* Odds ratio adjusted by age and gender.

Our results in general sheds light on the fact that longer *UGT1A1* repeat, increases the risk of developing hyperbilirubinemia and cholelithiasis irrespective of disease status. The coinheritance of *UGT1A1* polymorphism (7/7 or 6/7 repeat) and SCA was found to potentially increase the risk of developing cholelithiasis *(OR = 5.44).* We further assessed the global frequency of the genotype distribution of *UGT1A1* promoter polymorphism according to the location on the various continents (Table 5) [9], [11]–[35]. When comparing the Kuwaiti population with the Yemenis *(p = 0.448),* the origin of all Arabs and other Arabic population revealed no significant difference. Similarly, no significant difference was found between the Kuwaitis and the Caucasian populations *(p>0.05).* A highly significant differences was found between Kuwaitis, Asians, Africans, southern Americans and the pacific islands (Papua New Guinea Tonga Fiji) populations *p<0.05* [9].

**Table 5:** Global distribution of the *UGT1A1* promoter TATA box polymorphism.

Continent	Country	Total	6/6(%)	6/7(%)	7/7(%)	4/4(%)	5/5(%)	5/6(%)	5/7(%)	5/8(%)	6/8(%)	7/8(%)	8/8(%)	Ref
Europe	Iceland	69	29 (42)	33 (47.8)	7 (10.1)									9
	Uk	59	30 (50.8)	26 (44)	3 (5)									9
	Uk2	81	10 (12.3)	26 (32)	18 (22.2)		4 (5)	13 (16.2)	4 (5)		1 (1.2)	5 (6)		12
	Greek cypriots	47	23 (48.9)	19 (40.4)	4 (8.5)							1 (2.1)		9
	Greek 2	152	74 (48.7)	51 (33.5)	27 (17.8)									13
	Greek 3	37	18 (48.6)	12 (32.4)	7 (18.9)									14
	Basque	27	13 (48)	10 (37)	4 (14.8)									9
	Catalan	46	14 (30)	29 (63)	3 (6.5)									9
	Austria	255	94 (37)	127 (50)	33 (13)									15
	Croatia	1109	210 (19)	292 (26.3)	600 (54)				4 (0.36)		1 (0.09)	2 (0.18)		16
	Italian	98	43 (43.9)	39 ( 39.8)	16 (16.3)									17
	Slovenian	236	90 (38.1)	113 (47.9)	32 (13.6)									18
	Southern Germany	265	112 (42.3)	121 (45.7)	32 (12)									19
	Caucasian Germany	100	50 (50)	42 (42)	8 (8)									20
	Dutch Caucasian	430	190(44.2)	188(43.7)	51(11.9)									21
	Netherlands	41	17 (41)	18 (44)	6 (15)									22
Africa and African Origin	Kenya (Luo)	81	17 (19.1)	32 (35.9)	16 (17.9)		1 (1.12)	5 (5.6)	7 (7.8)	2 (2.2)				9
	Malawi	76	14 (18.9)	35 (47.2)	7 (9.4)			8 (10.8)	5 (6.7)	1 (1.3)	4 (5.4)	2 (2.7)		9
	Ivory coast	74	17 (22.9)	26 (35.1)	6 (8.1)			3 (4)	5 (6.7)	1 (1.3)	6 (8.1)	10 (13.5)		9
	Jamaica	72	22 (30.5)	22 (30.5)	7 (9.7)		2 (2.7)	8 (11.1)	5 (6.9)	1 (1.3)	2 (2.7)	2 (2.7)	1 (1.3)	9
	Madagascar	67	40 (59.7)	19 (28)	4 (5.9)			3 (4.4)				1 (1.4)		9
	Nigeria	226	28 (12.6)	88 (39.6)	45 (20.3)		2 (0.9)	26 (11.7)	15 (6.8)	1 (0.5)	5 (2.3)	11 95)	1 (0.5)	23
Asia	Hong Kong	50	38 (76)	11 (22)	1 (2)									9
	China Shanghai	1035	838 (81)	172 (16.6)	25 (2.4)									24
	Japan	36	35(97.2)	1 (2.8)										25
	Thailand	76	60 (78.9)	14 (18.4)	2 (2.6)									9
	Indonesia	60	40 (66.6)	17 (28.3)	3 (5)									9
	Korea	20	17 (85)	2 (10)	1 (5)									26
	Vietnam	83	70 (84.3)	12 (14.4)	1 (1.2)									9
	India 1	119	45 (37.8)	51 (42.8)	23 (19.3)									9
	India 2	50	25 (50)	21 (42)	4 (8)									27
	India 3	95	32 (33.6)	53 (55.7)	10 (10.5)									28
	Srilanka	229	58 (25.3)	116 (50.6)	55 (24)									9
	Bangladesh	26	6 (23)	15 (57.6)	5 (19.2)									9
	Myanmar	32	22 (68.75)	9 (28.12)	1 (3.1)									9
	Turkey	32	18 (56)	11 (34)	3 (10)									29
	Lebanon	42	16 (38)	22 (52.3)	4 (9.5)									9
	Egyptian	50	56 (64.8)	36 (38.4)	8 (6.8)									19
	Yemen	61	33 (54)	25 (40.9)	3 (4.9)									9
	Kuwait	62	26 (42)	34 (55)	2 (3)									*
South America	Amerindians	59	33 (55.9)	18 (30.5)	7 (11.8)							1 (1.6)		9
	Brazil													30
	Caucasians	71	32 (45)	28 (39)	9 (12.6)			1 (1.4)			1 (1.4)			30
	African derived	54	13 (24)	25 (46)	9 (16)			5 (10)	1 (2)	1 (2)				30
	Parakana Indians	32	12 (38)	19 (59)	1 (3)									30
North America	North Carolina	101	37 (36.6)	39 (38.6)	13 (12.9)		1 (0.9)	3 (2.9)	1 (0.9)		3 (2.9)	4 (3.9)		31
	North Carolina 2	200	56 (28)	72 (36)	33 (16.5)		1 (0.5)	19 (9.5)	10 (5)		4 (2)	5 (2.5)		32
	Mexico	375	155 (41.3)	174 (46.4)	38 (10.1)			7 (1.9)	1 (0.3)					33
	Boston (African American)	609	276 (45.3)	272 (44.7)	60 (9.8)									34
	New York	32	9 (28.25)	11 (34.25)	2 (6.25)		2 (6.25)	4 (12.5)	2 (6.25)			2 (6.25)		35
Pacific	Papua New Guinea	105	102 (97.1)	3 (2.8)	0 (0)									9
	Tonga	41	32 (78)	8 (19.5)	1 (2.4)									9
	Fiji	16	15 (93.7)	1 (6.25)	0 (0)									9
Chimpanzees		35				35 (100)								9

* current study

## Discussion

The theory of single gene disorders is no more accurate as the mutation in the globin genes alone is not sufficient to account for one of the major characteristics of RBC disorders such as bTH and SCA manifestations. There is a wide range of phenotypic expression of the disease, even in patients with identical hemoglobin genotypes from apparently similar environments [36]. This strongly suggests that modifier genes other than beta globin gene play a role in the phenotypic diversity within bTH, SCA as well as other hemoglobinopathies. We have recruited patients with bTH and SCA, followed their clinical data and hematologic/biochemical parameters for almost one to five year. The medical follow-up of SCA patients included the hepatobiliary ultrasound scan to assess the cholelithiasis.

Several risk factors for cholelithiasis have been identified in previous studies, one of which is the high total [37], [38] and unconjugated bilirubin concentrations [38], [39]. In our study, cholelithiasis was associated with high total bilirubin concentrations in SCA. No variation was noticed in terms of age and gender. We further evaluated the contribution of *UGT1A1* genotype to unconjugated hyperbilirubinemia and to the prevalence of cholelithiasis. Three (TA6, TA7 and TA8) of the four known alleles in the *UGT1A1* promoter region and four (TA6/6, TA6/7, TA6/8 and TA7/7) of the six possible genotypes were retrieved. We compared patients with and without cholelithiasis and found that the frequency of *UGT1A1* alleles (TA)6 was lower, and that of alleles (TA)7 was significantly higher in patients with cholelithiasis *(p = 0.002, OR = 2.74, 95% CI 1.42–5.25).* These data suggest a possible relationship between the *UGT1A1* locus and the occurrence of cholelithiasis in patients with SCA and bTH and most probably other hemoglobinopathies. Few studies have shown that allele (TA)7 is associated with high steady state concentrations of unconjugated bilirubin in serum and is a risk factor for cholelithiasis in adults with SCA or bTH. Furthermore, the importance of the pharmacogenomic effect of *UGT1A1* polymorphism was shown in modulating the hematologic response to hydroxyurea treatment in SCA patients [40]. Our study included patients with bTH and SCA and confirmed genetic risk factors for cholelithiasis in SCA, provided information about all the *UGT1A1* genotypes identified and strongly suggested that *UGT1A1* promoter polymorphism is a significant non-globin genetic modifier in these cases.

Two global surveys showed a wide variation in the frequency of the number of (TA) repeats in the promoter region of the *UGT1A1* gene [9], [41]. We have further aimed to update the survey and compared the data obtained from Kuwaiti population with other ethnicities (table 5). (TA)6 is the most common allele in all studied populations whereas (TA)7 is the highest in the Croatian population where more than half (54.1%) of this population have homozygous (TA)7/7. The Srilankian (24%), Bangladesh (19.2%) and a subpopulation of the India (19.3%) also have a high frequency of homozygous (TA)7/7. A subset of the UK population (22.2%) and the Nigerian population (20.3) has a high frequency of homozygous (TA)7/7. Allele (TA)7 was rarest in Pacific islands (0%) (Papua New Guinea and Fiji) and also in Asians particularly Vietnamese and Chinese populations while has a highly variable frequency in European populations. In African populations, the spectrum is quite broad, ranging from five to eight (TA) repeats. The Kuwaiti population was the closest to other Arabic populations and to the Caucasians. A highly significant differences was found between Kuwaitis and Asians, Africans (Nigerian, Merganser, Kenya), southern Americans and the pacific islands (Papua New Guinea Tonga, Fiji) populations p<0.05. However, Africans Malawai and the Jamaicans have not shown significant difference with the Arab and Caucasian populations.

## Materials and Methods

A total of 270 subjects were recruited in the study (116 SCA, 92 homozygous bTH and 62 health controls) after obtaining informed consent. Of these, 236 subjects who fit the selection criteria were carefully selected. All subjects were adult (>26 years of age) Kuwaiti Arabs with 1∶1.5 male to female ratios. The patients were regularly transfused with packed red cells every 4 weeks to maintain mean hemoglobin levels above 120 g/l, and were receiving regular iron chelating therapy (deferoxamine mesylate 40 mg/kg daily). Blood was withdrawn from the patients before receiving transfusion. Healthy Subjects were excluded if they had a past or present history of hepatic/hematological disease. None of the recruited bTH and SCA subjects had a history of primary hepatic disorders, excessive alcoholism, chronic use of medications or narcotics, nor had received any drug two weeks prior to our investigation. Clinical characteristics of each subject selected for the study is detailed in table 6. Fifty nine percentage of the recruited hemoglobinopathy cases underwent liver/biliary ultrasound scans (104/174) to assess the cholelithiasis. A 67.3% of 104 SCA patients had gallstone disease. Written informed consent was obtained from each participant, under the protocols approved by the Joined Committee for the Protection of Human Subjects in Research.

**Table 6:** Characteristics of study subjects.

Factors	bTH (n = 70)	SCA (n = 104)	HC (n = 62)
Age	29.37±2.47	31.24±4.93	30.58±5.75
Gender (male)	37% (26)	47% (48)	35.5% (22)
Ethnicity	Arabs		
WBC (×10^9^/l)	7.89±3.04	8.79±3.45	8.56±1.79
RBC(×10^12^/l)	4.10±0.81	3.99±0.98	4.23±0.308
Hb level(g/l)	124±5.62	126±18.9	128±18.9
Gall stone	–	67.3% (70)	–

bTH- b-thalassemia, SCA- Sickle cell Anaemia, HC- Healthy control.

### Measurement of Serum Bilirubin Levels

After an overnight fast, Blood and serum samples were collected from each subject in EDTA treated and plain tubes respectively. Plasma concentrations of total bilirubin were determined by diazo method at least three times within 6 months. Mean values were considered for the comparative study. Liver function tests were performed in the hospital laboratory by a standardized colorimetric procedure LXI, Beckman biochemical analyzer.

### Genotyping of UGT1A1 (TA)n Promoter Polymorphism

DNA was extracted from the blood samples using the QIAamp Mini DNA extraction kit (QIAGEN, Germany). The forward primer 5′-GAGGTTCTGGAAGTACTTTGC-3′ and the reverse primer 5′-CCAAGCATGCTCAGCCAG-3′ were used to amplify the region of interest in the 5′ region of the UGT1A1 promoter fragment of 409 bp. The PCR amplicons were subjected to dHPLC (denaturing high-performance liquid chromatography) to screen for the number of repeats in the TATA box. The presence of two well-resolved peaks reveals the heterozygous condition such as (TA)6/(TA)7. The presence of one peak is characteristic of the homozygous condition. To distinguish (TA)6 from (TA)7 homozygotes, each sample showing a single peak was mixed with (TA)6/(TA)6 control DNA under conditions allowing heteroduplex formation. The homozygous condition for (TA)7 was revealed by a double peak, whereas for (TA)6 homozygotes, there was no change in the chromatogram showing a single peak. Another screening method we used was the fragment analysis where the forward primer used was labeled with FAM fluoresce dye and amplicons were subjected to capillary electrophoresis. Genotyping results for 60 randomly selected samples (25% of tested cases) were further confirmed by sequencing using genetic analyzer ABI 3100 (Applied BioSystem, USA). The dHPLC results showed a 100% match with those obtained by direct sequencing and fragment analysis.

### Statistical Analysis

Data collection, data management and statistical analysis were performed with SPSS 19.0 (SPSS, Inc., Chicago, IL). Allele and genotype frequencies of *UGT1A1* promoter polymorphism were calculated manually for each subjects. Observed genotypes showed no significant deviation (*p*>0.05) from Hardy Weinberg equilibrium by Genepop software. Differences in the genotypic and the allelic frequency between the patients and controls were assessed by the chi-squared *p* value test. A p-value of <0.05 was considered to be statistically significant. Average serum total bilirubin is expressed as mean values ± Standard deviation (unless stated otherwise). Test for homogeneity (p<0.05) indicated asymmetric distribution, hence the total bilirubin data was log transformed and the statistical difference between the compared subgroups were assessed either by one-way ANOVA test or students t-test. Association of genotypes and total serum bilirubin level to disease status were assessed by binary logistic regression adjusting for factors such as gender and age.

## Conclusion

The frequency of *UGT1A1* (TA)*_n_* promoter polymorphism genotypes was determined for the first time in healthy Kuwaiti population, and is similar to frequencies observed in Caucasian populations. The extremely rare (TA)_8_ allele in Caucasians were also found in Kuwaitis. We further reveal that bTH and SCA patients (regardless of the age or gender) can be classified into three risk groups according to *UGT1A1* genotype. Patients homozygous for (TA)7 are associated with a high frequency of cholelithiasis. *UGT1A1* genotyping is therefore a potentially useful tool for identifying individuals with hemoglobinopathy at high risk of cholelithiasis and requiring close clinical monitoring.

## References

[1] Bosma PJ, Seppen J, Goldhoorn B, Bakker C, Oude Elferink RP, et al (1994) Bilirubin UDP-glucuronosyltransferase 1 is the only relevant bilirubin glucuronidating isoform in man. J Biol Chem 269: 17960–17964. 8027054

[2] Schubert TT (1987) Hepatobiliary system in sickle cell disease. Gastroenterology 90: 2013–21. 10.1016/0016-5085(86)90276-3 3516788

[3] Sampietro M, Iolascon A (1999) Molecular pathology of Crigler–Najjar type I and II and Gilbert’s syndromes. Haematologica 84: 150–157. 10091414

[4] Bosma PJ (2003) Inherited disorders of bilirubin metabolism. J Hepatol 38: 107–117. 12480568 10.1016/s0168-8278(02)00359-8

[5] Clarke DJ, Moghrabi N, Monaghan G, Cassidy A, Boxer M, et al (1997) Genetic defects of the UDP-glucuronosyltransferase-1 (UGT1) gene that cause familial non-haemolytic unconjugated hyperbilirubinaemias. Clin Chim Acta 266: 63–74. 9435989 10.1016/s0009-8981(97)00167-8

[6] Bosma PJ, Chowdhury JR, Bakker C, Gantla S, de Boer A, et al (1995) The genetic basis of the reduced expression of bilirubin UDP-glucuronosyltransferase 1 in Gilbert’s syndrome. N Engl J Med 333: 1171–1175. 7565971 10.1056/NEJM199511023331802

[7] Monaghan G, Ryan M, Seddon R, Hume R, Burchell B (1996) Genetic variation in bilirubin UDP-glucuronosyltransferase gene promoter and Gilbert’s syndrome. Lancet 347: 578–581. 8596320 10.1016/s0140-6736(96)91273-8

[8] Beutler E, Gelbart T, Demina A (1998) Racial variability in the UDP-glucuronosyltransferase 1 (UGT1A1) promoter: a balanced polymorphism for regulation of bilirubin metabolism. Proc Natl Acad Sci U S A 95: 8170–8174. 9653159 10.1073/pnas.95.14.8170 PMC20948

[9] Premawardhena A, Fisher CA, Liu YT, Verma IC, de Silva S, et al (2003) The global distribution of length polymorphisms of the promoters of the glucuronosyltransferase 1 gene (UGT1A1): hematologic and evolutionary implications. Blood Cells Mol Di. 31: 98–101. 10.1016/s1079-9796(03)00071-8 12850492

[10] Haberkern CM, Neumayr LD, Orringer EP, Earles AN, Robertson SM, et al (1997) Cholecystectomy in sickle cell anemia patients: perioperative outcome of 364 cases from the National Preoperative Transfusion Study. Preoperative Transfusion in Sickle Cell Disease Study Group. Blood 89: 1533–42. 9057634

[11] Samilchuk E, Al-Suliman I, Usanga E, Al Awadi S (2003) Glucose-6-phosphate dehydrogenase (G6PD) mutations and UDP-glucuronosyltransferase promoter polymorphism among G6PD deficient Kuwaitis. Blood Cells Mol Dis 31: 201–5. 12972027 10.1016/s1079-9796(03)00125-6

[12] Vasavda N, Menzel S, Kondaveeti S, Maytham E, Awogbade M, et al (2007) The linear effects of a-thalassaemia, the UGT1A1 and HMOX1 polymorphisms on cholelithiasis in sickle cell disease. British Journal of Haematology 138: 263–270. 17593033 10.1111/j.1365-2141.2007.06643.x

[13] Tsezou A, Tzetis M, Giannatou E, Spanos I, Roma E, et al (2009) Gilbert syndrome as a predisposing factor for cholelithiasis risk in the Greek adult population. Genet Test Mol Biomarkers 13: 143–6. 19309288 10.1089/gtmb.2008.0095

[14] Kalotychou V, Antonatou K, Tzanetea R, Terpos E, Loukopoulos D, et al (2003) Analysis of the A(TA)(n)TAA configuration in the promoter region of the UGT1 A1 gene in Greek patients with thalassemia intermedia and sickle cell disease. Blood Cells Mol Dis 31: 38–42. 12850481 10.1016/s1079-9796(03)00118-9

[15] Rantner B, Kollerits B, Anderwald-Stadler M, Klein-Weigel P, Gruber I, et al (2008) Association between the *UGT1A1* TA-Repeat Polymorphism and Bilirubin Concentration in Patients with Intermittent Claudication: Results from the CAVASIC Study Clinical. Chemistry 54: 851–857. 10.1373/clinchem.2007.102046 18375480

[16] Nikolac N, Simundic A, Topic E, Jurcic Z, Stefanovic M, et al (2008) Rare TA repeats in promoter TATA box of the UDP glucuronosyltransferase (*UGT1A1*) gene in Croatian subjects. Clin Chem Lab Med 46: 174–178. 18324905 10.1515/CCLM.2008.035

[17] Biondi ML, Turri O, Dilillo D, Stival G, Guagnellini E (1999) Contribution of the TATA-Box Genotype (Gilbert Syndrome) to Serum Bilirubin Concentrations in the Italian Population. Clinical Chemistry 45: 897–898. 10352000

[18] Ostanek B, Furlan D, Mavec T, Lukac-Bajalo J (2007) UGT1A1 (TA)n promoter polymorphism–A new case of a (TA)8 allele in Caucasians. Blood Cells Molecules and Diseases 38: 78–82. 10.1016/j.bcmd.2006.10.160 17196409

[19] Borlak J, Thum T, Landt O, Erb K, Hermann R (2000) Molecular Diagnosis of a Familial Nonhemolytic Hyperbilirubinemia (Gilbert’s Syndrome) in Healthy Subjects. Hepatology 32: 792–795. 11003624 10.1053/jhep.2000.18193

[20] Kohlea C, Mohrlea B, Munzela PA, Schwabb M, Wernetc D, et al (2003) Frequent co-occurrence of the TATA box mutation associated with Gilbert’s syndrome (UGT1A1*28) with other polymorphisms of the UDP-glucuronosyltransferase-1 locus (UGT1A6*2 and UGT1A7*3) in Caucasians and Egyptians. Biochemical Pharmacology 65: 1521–1527. 12732365 10.1016/s0006-2952(03)00074-1

[21] Te Morsche RH, Zusterzeel PL, Raijmakers MT, Roes EM, Steegers EA, et al (2001) Polymorphism in the promoter region of the bilirubin UDP–glucuronosyltransferase (Gilbert’s syndrome) in healthy Dutch subjects. Hepatology 33: 765. 11230763 10.1053/jhep.2001.0103303le03

[22] Raijmakers M, Jansen P, Steegers E, Peters W (2000) Association of human liver bilirubin UDP-glucuronyltransferase activity with a polymorphism in the promoter region of the UGTlAl gene. J Hepatol 33: 348–35. 11019988 10.1016/s0168-8278(00)80268-8

[23] Huo D, Kim H, Adebamowo CA, Ogundiran TO, Akang E, et al (2008) Genetic polymorphisms in uridine diphosphoglucuronosyltransferase 1A1 and breast cancer risk in Africans. Breast Cancer Res Treat 110: 367–376. 17909964 10.1007/s10549-007-9720-7 PMC4384416

[24] Deming S, Zheng W, Xu W, Cai Q, Ruan Z, et al (2008) UGT1A1 Genetic Polymorphisms, Endogenous Estrogen Exposure, Soy Food Intake, and Endometrial Cancer Risk Cancer Epidemiol. Biomarkers Prev 17: 563–70. 10.1158/1055-9965.EPI-07-0752 18349273

[25] Sugatani J, Mizushima K, Osabe M, Yamakawa K, Kakizaki S, et al (2008) Transcriptional regulation of human UGT1A1 gene expression through distal and proximal promoter motifs: implication of defects in the UGT1A1 gene promoter. Naunyn-Schmiedeberg’s Arch Pharmacol 377: 597–605. 18172616 10.1007/s00210-007-0226-y

[26] Ki CS, Lee KA, Lee SY, Kim HJ, Cho SS, et al (2003) Haplotype Structure of the UDP-Glucuronosyltransferase 1A1 (*UGT1A1*) Gene and Its Relationship to Serum Total Bilirubin Concentration in a Male Korean Population. Clinical Chemistry 49: 2078–2081. 14633881 10.1373/clinchem.2003.024174

[27] Agrawal S, Kumar P, Rathi R, Sharma N, Das R, et al (2009) *UGT1A1* Gene Polymorphisms in North Indian Neonates Presenting with Unconjugated Hyperbilirubinemia. Pediatr Res 65: 675–680. 19430380 10.1203/PDR.0b013e31819ed5de

[28] Farheen S, Sengupta S, Santra A, Pal S, Dhali GK, et al (2006) Gilbert’s syndrome: High frequency of the (TA)7 TAA allele in India and its interaction with a novel CAT insertion in promoter of the gene for bilirubin UDP-glucuronosyltransferase 1 gene. World J Gastroenterol 12: 2269–2275. 16610035 10.3748/wjg.v12.i14.2269 PMC4087660

[29] Babaoglu M, Yigit S, Aynacioglu AS, Kerb R, Yurdakok M, et al (2006) Neonatal Jaundice and Bilirubin UDP-Glucuronosyl Transferase 1A1 Gene Polymorphism in Turkish Patients. Basic Clin Pharmacol Toxicol 98: 377–380. 16623861 10.1111/j.1742-7843.2006.pto_341.x

[30] Fertrin KY, Gonçalves MS, Saad ST, Costa FF (2002) Frequencies of UDP-glucuronosyltransferase 1 (UGT1A1) gene promoter polymorphisms among distinct ethnic groups from Brazil. Am J Med Genet 108: 117–9. 11857560

[31] Grant D, Hall I, Eastmond D, Jones I, Bell D (2004) Bilirubin UDP-glucuronosyltransferase 1A1 (*UGT1A1*) gene promoter polymorphisms and *HPRT*, glycophorin A, and micronuclei mutant frequencies in human blood. Mutation Research 560: 1–10. 15099818 10.1016/j.mrgentox.2004.01.010

[32] Guillemette C, Millikan R, Newman B, Housman D (2000) Genetic Polymorphisms in Uridine Diphospho-Glucuronosyltransferase 1A1 and Association with Breast Cancer among African Americans1. Cancer Research 60: 950–956. 10706110

[33] Arambula E, Vaca G (2002) Genotyping by Cold Single-Strand Conformation Polymorphism of the *UGT1A1* Promoter Polymorphism in Mexican Mestizos. Blood Cells Molecules and Diseases 28: 86–90. 10.1006/bcmd.2001.0481 11987245

[34] Guillemette C, De Vivo I, Hankinson SE, Haiman CA, Spiegelman D, et al (2001) Association of Genetic Polymorphisms in UGT1A1 with Breast Cancer and Plasma Hormone Levels1. Cancer Epidemiol Biomarkers Prev 10: 711–4. 11401924

[35] Huang C, Dulau A, Su-Rick C, Pan Q (2007) Validation of Rapid Polymerase Chain Reaction-based. Detection of All Length Polymorphisms in the UGT 1A1 Gene Promoter. Diagn Mol Pathol 16: 50–53. 17471158 10.1097/01.pdm.0000213467.91139.c9

[36] Serjeant GR, Serjeant BE (1993) Management of sickle cell disease; lessons from the Jamaican Cohort Study. Blood Rev 7: 37–45. 10.1016/0268-960x(93)90001-k 8241829

[37] Sarnaik S, Slovis TL, Corbett DP, Emami A, Whitten CF (1980) Incidence of cholelithiasis in sickle cell anemia using the ultrasonic gray-scale technique. J Pediatr 96: 1005–8. 7373460 10.1016/s0022-3476(80)80626-3

[38] McCall IW, Desai P, Serjeant BE, Serjeant GR (1977) Cholelithiasis in Jamaican patients with homozygous sickle cell disease. Am J Hematol 3: 15–21. 602934 10.1002/ajh.2830030102

[39] Webb DK, Darby JS, Dunn DT, Terry SI, Serjeant GR (1989) Gall stones in Jamaican children with homozygous sickle cell disease. Arch Dis Child 64: 693–6. 2658854 10.1136/adc.64.5.693 PMC1792039

[40] Heeney MM, Howard TA, Zimmerman SA, Ware RE (2003) UGT1A promoter polymorphisms influence bilirubin response to hydroxyurea therapy in sickle cell anemia. J Lab Clin Med 141: 279–82. 12677174 10.1067/mlc.2003.28

[41] Hall D, Ybazeta G, Destro-Bisol G, Petzl-Erler ML, Di Rienzo A (1999) Variability at the uridine diphosphate glucuronosyltransferase 1A1 promoter in human populations and primates. Pharmacogenetics 9: 591–9. 10591539

